# A Novel System for Supporting Autism Diagnosis Using Home Videos: Iterative Development and Evaluation of System Design

**DOI:** 10.2196/mhealth.4393

**Published:** 2015-06-17

**Authors:** Nazneen Nazneen, Agata Rozga, Christopher J Smith, Ron Oberleitner, Gregory D Abowd, Rosa I Arriaga

**Affiliations:** ^1^ Georgia Institute of Technology Atlanta, GA United States; ^2^ Southwest Autism Research & Resource Center Phoenix, AZ United States; ^3^ Behavior Imaging Solutions Boise, ID United States

**Keywords:** asynchronous telemedicine system, in-home behavior recording, naturalistic observation diagnostic assessment, NODA Connect, NODA smartCapture, remote autism diagnosis

## Abstract

**Background:**

Observing behavior in the natural environment is valuable to obtain an accurate and comprehensive assessment of a child’s behavior, but in practice it is limited to in-clinic observation. Research shows significant time lag between when parents first become concerned and when the child is finally diagnosed with autism. This lag can delay early interventions that have been shown to improve developmental outcomes.

**Objective:**

To develop and evaluate the design of an asynchronous system that allows parents to easily collect clinically valid in-home videos of their child’s behavior and supports diagnosticians in completing diagnostic assessment of autism.

**Methods:**

First, interviews were conducted with 11 clinicians and 6 families to solicit feedback from stakeholders about the system concept. Next, the system was iteratively designed, informed by experiences of families using it in a controlled home-like experimental setting and a participatory design process involving domain experts. Finally, in-field evaluation of the system design was conducted with 5 families of children (4 with previous autism diagnosis and 1 child typically developing) and 3 diagnosticians. For each family, 2 diagnosticians, blind to the child’s previous diagnostic status, independently completed an autism diagnosis via our system. We compared the outcome of the assessment between the 2 diagnosticians, and between each diagnostician and the child’s previous diagnostic status.

**Results:**

The system that resulted through the iterative design process includes (1) NODA smartCapture, a mobile phone-based application for parents to record prescribed video evidence at home; and (2) NODA Connect, a Web portal for diagnosticians to direct in-home video collection, access developmental history, and conduct an assessment by linking evidence of behaviors tagged in the videos to the Diagnostic and Statistical Manual of Mental Disorders criteria. Applying clinical judgment, the diagnostician concludes a diagnostic outcome. During field evaluation, without prior training, parents easily (average rating of 4 on a 5-point scale) used the system to record video evidence. Across all in-home video evidence recorded during field evaluation, 96% (26/27) were judged as clinically useful, for performing an autism diagnosis. For 4 children (3 with autism and 1 typically developing), both diagnosticians independently arrived at the correct diagnostic status (autism versus typical). Overall, in 91% of assessments (10/11) via NODA Connect, diagnosticians confidently (average rating 4.5 on a 5-point scale) concluded a diagnostic outcome that matched with the child’s previous diagnostic status.

**Conclusions:**

The in-field evaluation demonstrated that the system’s design enabled parents to easily record clinically valid evidence of their child’s behavior, and diagnosticians to complete a diagnostic assessment. These results shed light on the potential for appropriately designed telehealth technology to support clinical assessments using in-home video captured by families. This assessment model can be readily generalized to other conditions where direct observation of behavior plays a central role in the assessment process.

## Introduction

### Background and Motivation

According to the Centers for Disease Control and Prevention, the prevalence of autism in the United States has been increasing dramatically, from 1 in 150 to 1 in 68 children between 2000 and 2010 [1,2]. Over the same period, the median age of diagnosis remained relatively stable, around 53 months [1,2].

One key challenge with respect to diagnosing autism is the significant time lag (20-60 months) between the age at which parents first become concerned about their child’s development and the age at which the child receives a diagnosis from a qualified professional [3-5]. Moreover, many ethnic minorities, low-income families, and rural communities lack access to health care professionals with autism-specific expertise, resulting in delays in diagnosis [6-9]. Even in urban communities where services are more widely available, timely access to diagnostic services is often hampered by long waiting lists. Delays in diagnosis can lead to delays in early intervention services that have been shown to improve future learning capabilities and developmental outcomes [10-13].

Another key challenge with respect to diagnosing autism is that although clinical professionals acknowledge that observing behavior in the natural environment (eg, the home) is preferred for a comprehensive assessment, in practice behavioral observations are limited to a single in-clinic observation [10,11,14]. There are various barriers to more widespread use of home-based observation [14-18]. It is time consuming and resource intensive for clinicians to travel to each family’s home to conduct an observation, and impractical to do so for remotely located families. Even when home visits are feasible, the presence of an unfamiliar observer may cause children to alter their behavior due to their awareness of being observed. Such reactivity poses a threat to the validity of any data that are collected. In addition, child behaviors of interest may not occur during the short span of a clinician’s home visit.

In this paper, we consider the opportunity of designing a telehealth solution to support remote diagnosis of autism through parent-recorded behavioral evidence of child suspected to have autism in the home. Telehealth technology can connect families with clinicians and accelerate the diagnostic process. Indeed, such technologies have recently been investigated as a means of supporting the delivery of treatment for individuals with autism spectrum disorder, including remote coaching of parent-implemented early intervention programs [19-21], behavioral assessments [22,23], and professional development [24]. Few attempts have been made at exploring the potential for such technologies to support diagnostic assessments [25,26].

Most current telehealth technologies support a real-time interaction between a remotely located clinician and a caregiver or patient. By contrast, “store-and-forward” telehealth systems support video recordings of live events, which are subsequently shared with a clinical expert for review and assessment. The latter asynchronous approach, which we have adopted, offers several key advantages particularly relevant to the case of remote diagnosis of autism. It enables families to record videos in their home, in the course of their day-to-day activities, which ensures the capture of natural expressions of child behavior that are widely acknowledged as crucial to making an accurate and comprehensive diagnostic assessment [10,11,14]. Moreover, because home recordings can be carried out over the course of several days, they may mitigate some of the consequences of a single clinic-based or live telehealth assessment. These include the child’s reactivity, child’s current mood or level of fatigue, or the likelihood that low-frequency behaviors may not be observed. From a practical standpoint, it minimizes the need to coordinate schedules with a clinician, and reduces the need for remotely located families to travel long distances to a clinic.

### Research Questions and Contribution

Our research addresses two key challenges in designing a system for remote autism diagnosis using home videos. Perhaps the most important challenge is how to enable parents to easily record clinically relevant video evidence of their child’s behavior. Diagnostic assessments are typically designed to enable the diagnostician to observe the child under more or less structured periods and different situations for a rich sampling of the child’s behavior. Parents can record and share their concerns about their child, but may not know the specific types of situations and behaviors the diagnostician needs to observe. Thus, the first research challenge is how the parent-recorded video can be turned into meaningful clinical evidence through the use of the right kind of technology and the design of the right user experience with that technology. This paper summarizes our work on identifying and evaluating specific design features that contribute to ease of use of the in-home recording system and clinical validity of parent-recorded video evidence. The second challenge involves supporting diagnosticians in completing the remote autism diagnosis. This involves enabling diagnosticians to review the videos in a systematic and structured way so that they can map the situations and behaviors that they observe in the video to the Diagnostic and Statistical Manual of Mental Disorders (DSM) diagnostic criteria [27,28]. In other words, the parent-recorded video of child behavior must produce observations that become evidence to support clinical judgment. This paper summaries our work on identification and evaluation of specific design features that support diagnosticians in completing a remote diagnostic assessment.

The system that resulted from this work includes two components: NODA smartCapture and NODA Connect. NODA smartCapture is a mobile phone-based application that enables parents to easily record clinically relevant prescribed video evidence of their child’s behavior. It supports recording and uploading 4, up to 10-minute long naturalistic observation diagnostic assessment (NODA) scenarios, that were chosen based on pilot research on video-based diagnosis of autism [29]. These scenarios include (1) the child playing alone, (2) the child playing with a sibling or peer, (3) a family mealtime, and (4) any behavior that is of concern to the parent. The first 3 scenarios provide opportunities for typical social communication and play-based behaviors, whereas the last scenario allows parents to share evidence of a behavior that is of particular concern to them. The NODA Connect is a Web portal for diagnosticians to direct in-home video collection, access the child’s developmental history, and conduct a remote diagnostic assessment by linking evidence of behaviors tagged in the videos to DSM criteria. Relying on clinical judgment, the diagnostician renders an opinion about the child’s diagnostic outcome.

### Paper Outline

The rest of this paper is structured as follows. We first describe interviews with relevant stakeholders about our system concept, that is, “remote assessment based on in-home video evidence.” Next, we outline the iterative design of NODA smartCapture and NODA Connect. We then describe an in-field evaluation, in which 5 families used NODA smartCapture in their homes to collect prescribed behavioral evidence and 2 diagnosticians reviewed videos from each family and independently performed an autism diagnosis using NODA Connect. We report results on ease of use of NODA smartCapture, clinical validity of recorded evidence, and NODA Connect’s support for diagnosticians in completion of a remote diagnostic assessment. We conclude with a discussion on the utility and limitations of the system, potential design enhancements, our vision for adoption of the system within current autism diagnostic practices, and how such a prescription, collection, and assessment model can be generalized to other clinical applications.

## Methods

### Overview

First, interviews were conducted with parents of children with autism and clinicians to seek input from these key stakeholders about the overall concept of the system. Next, NODA smartCapture was iteratively designed, informed by experiences of families using it in a controlled experimental home-like setting. The NODA Connect was designed using a participatory design process involving a collaborating diagnostician with 20 years of experience in autism diagnosis and a domain expert in autism. Finally, an in-field evaluation was conducted with families and diagnosticians.

### Stage 1: Insight from Stakeholders

We conducted a series of structured one-on-one interviews, each lasting 2 hours, with parents (n=7) of children with autism and clinicians (n=11) who work with this population. These interviews allowed us to gather input from these key stakeholders about the overall system concept of “remote assessment based on in-home video evidence,” and about its feasibility and potential utility. During the interview, a mock-up design based on previous studies [29,30] was presented to elicit feedback from stakeholders. The mock-up design included a mobile phone-based recording application to record the NODA scenarios and a Web-based assessment portal to review the videos and tag behaviors for assessment.

### Stage 2: Iterative Development

#### smartCapture Iterative development

**Figure 1:**
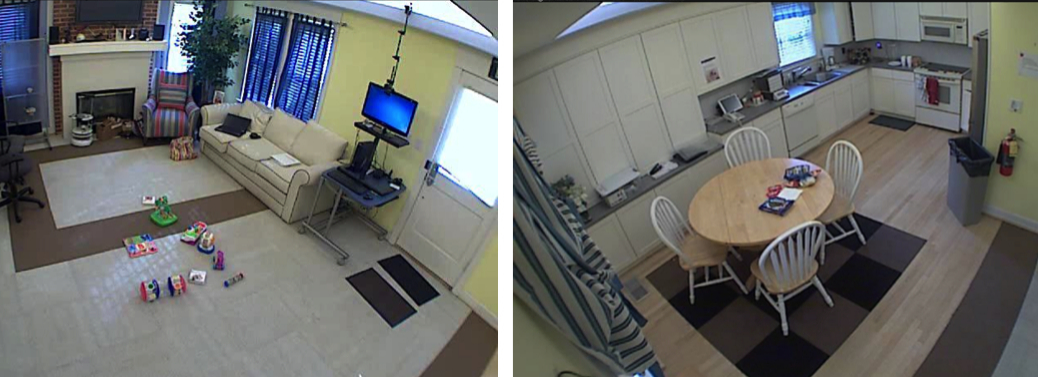
Aware Home setup for families to experience NODA smartCapture.

The NODA smartCapture application resulting from stage 1 was iteratively improved, informed by the experiences of families using it in a home-like experimental setup. Our goal was to identify specific design features that would enable parents to easily record clinically valid video evidence by analyzing the usage pattern of the recording application in a controlled setting. Families (n=8) and their child with autism as well as any siblings (n=18) visited the Georgia Tech Aware Home [31] for 2 hours, 1 family at a time. The Aware Home has the look of a single-family home (fully equipped kitchen; living room, bedrooms, and bathroom; furniture; TV, etc) except there are a number of cameras installed throughout, enabling both recording and viewing a live feed of interactions happening in different parts of the home (Figure 1).

Parents were asked to use the NODA smartCapture to record the 4 NODA scenarios, for up to 10 minutes each. Before the study, the Aware Home was set up with toys and items for the snack (to simulate the family mealtime). Ceiling-mounted cameras allowed us to observe the family from another room live as they were using NODA smartCapture, and to record the whole session for subsequent review.

After the video recording, each parent completed an interview and was asked to rate the ease of use of the system on a scale ranging from 1 (not easy to use) to 5 (very easy to use). In addition to this feedback from parents and the video evidence recorded by them using NODA smartCapture, video recordings from the fixed ceiling cameras were reviewed to gain further insight into how parents used the system.

The collaborating diagnostician was asked to rate each parent-collected video, for its clinical validity, on a scale of 0-2 and give a justification for the assigned rating. A rating of “0” means that the video is not clinically valid for conducting assessment whereas a rating of “2” indicates that the video is clinically valid. A rating of “1” indicates that the video is clinically valid but an additional video might be required to fully assess the associated scenario. By analyzing the collaborating diagnostician’s reasons for the assigned rating, we identified specific issues that lowered the clinical value of a video evidence.

After the first 4 of the total 8 families completed their participation, the design of NODA smartCapture was revised based on initial findings about ease of use and issues lowering clinical utility. The revised system was tested with the remaining 4 families. Once all families completed participation, NODA smartCapture was subsequently improved based on findings from the experience of the last 4 families.

#### NODA Connect Iterative Development

The NODA Connect Web portal was designed, through an iterative design process, for diagnosticians to direct in-home video-collection process and conduct remote diagnostic assessment. Our goal was to identify specific features that would support diagnosticians in completing the diagnostic assessment for autism based on parent-collected videos, developmental history information, and their clinical judgment. The initial design of NODA Connect was informed by previous pilot research [29] and feedback from stakeholders solicited during the Stage 1 interviews about the system concept. However, major design contributions came from a participatory design process involving a collaborating diagnostician who had 20 years of experience in conducting autism diagnosis and a domain expert in autism. Participatory design is a common method in the technology design community whereby the designer works closely with the target user to collaboratively iterate on the design of a technology [32]. Before the in-field evaluation in the final stage of the research, the design of the NODA Connect platform was further improved based on findings from a pilot assessment conducted via NODA Connect by the collaborating diagnostician.

### Stage 3: In-Field Evaluation

The iterative design process described in Stage 2 resulted in a final design of the remote autism diagnostic assessment system that was then evaluated in the field. During this evaluation, the parents used NODA smartCapture in their homes to record behavior evidence and the diagnosticians used NODA Connect to review and tag the videos, and to complete a diagnostic assessment. We recruited 4 families with at least 1 child with a previously confirmed diagnosis on the autism spectrum and 1 family with a typically developing child. Children were between 2 and 6 years of age (average 4 years). Parents were not given any prior training on NODA smartCapture. They were hand-delivered a kit that included NODA smartCapture preinstalled on an iPod touch and a tripod for mounting the iPod. During an in-home deployment that lasted an average of 2 weeks, each family was asked to complete a brief child developmental history online and use the NODA smartCapture application to record and upload the 4 10-minute NODA scenarios. The collaborating diagnostician remotely guided the in-home evidence-collection process by reviewing the videos as they were uploaded and sending alerts to the family as needed to request that they rerecord a particular scenario.

We recruited 3 diagnosticians experienced in autism diagnosis and unfamiliar with our system to complete the independent diagnostic assessments via NODA Connect. Each family’s videos were reviewed by at least 2 diagnosticians, who were blind to the diagnostic status of the child. After completing each diagnostic assessment via NODA Connect, the diagnosticians concluded whether the child had autism or was typically developing. They were prompted to assign confidence ratings to the diagnostic outcome: “How confident are you that the child has autism?” and “How confident are you that the child is typically developing?” on a scale from 1 (not confident) to 5 (extremely confident). Including both of these ratings allowed diagnosticians to indicate diagnostic uncertainty in cases where they were confident that the child does not have autism but also did not think the child was typically developing. Other than the child’s age, no other information was disclosed to the diagnosticians about the child’s developmental history until they completed the diagnostic assessment via NODA Connect and reached a decision about the child’s diagnostic outcome. At the end of this process, a follow-up interview was conducted for the diagnosticians to reflect on their experience of remote diagnostic assessment. The child’s previous diagnosis and developmental history were revealed during the interview.

Data analysis of the in-field evaluation of NODA smartCapture consisted of assessing its ease of use based on parent ratings, the quality of the videos recorded by parents, and the system log about parents’ reliance on help menu and navigation patterns though NODA smartCapture. The collaborating diagnostician rated the clinical utility of parent-recorded videos using the same scale as described earlier for Stage 2. Data analysis of the in-field evaluation of NODA Connect consisted of analysis of how diagnosticians completed diagnostic assessment by tagging videos and completing DSM checklist through NODA Connect. To conduct this analysis, videos of screen capture when diagnosticians were conducting assessment through NODA Connect were examined. In addition, for each child, we compared the outcome of the assessment between the 2 diagnosticians, and between each diagnostician and the child’s previous diagnostic status.

## Results

### Ease of Use of NODA smartCapture

**Figure 2:**
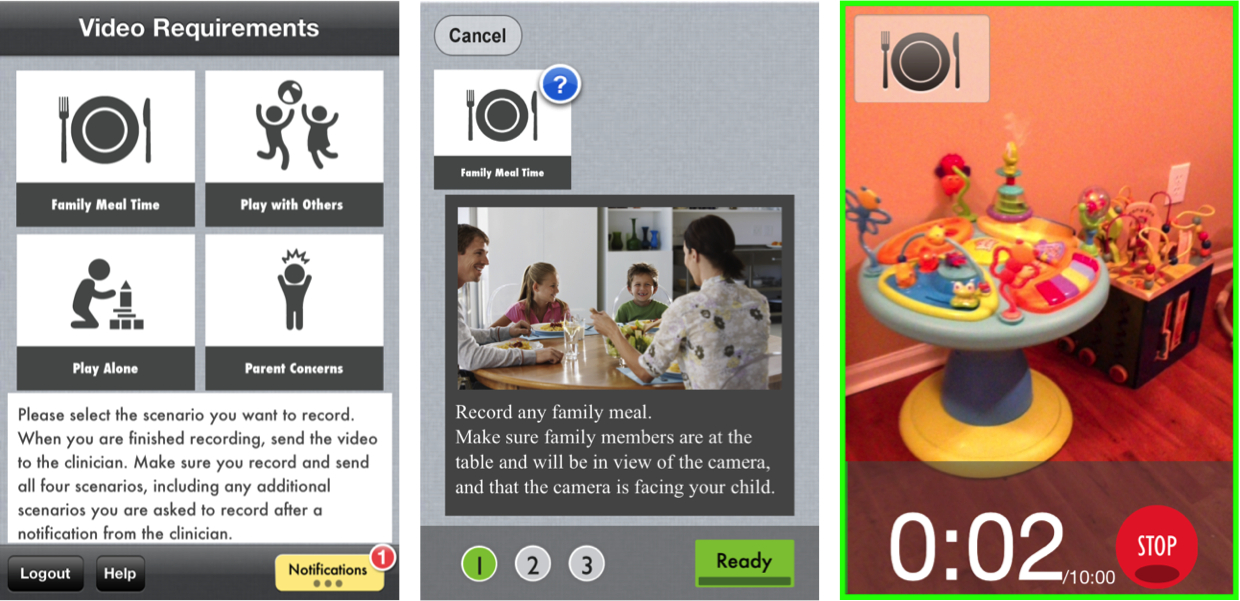
NODA smartCapture. (1) Home screen showing 4 NODA scenarios, as well as status of ones recorded. (2) Each scenario has recording instructions for parents as prescription. Pressing “Ready” proceeds to recording interface. (3) Recording mode with clear time-elapsed status and a green boundary to reinforce recording mode.

Based on experiments in the controlled home-like setting in Stage 2, three main features were added to NODA Connect to facilitate ease of use. First, icons on the home screen clearly depict each of the 4 NODA scenarios parents are being asked to record (Figure 2). Second, clear and redundant cues for the recording status were added so that a parent would know whether a video is being recorded or not, and how many minutes of recording have elapsed (Figure 2). Third, in addition to the “Stop Recording” button, an autostop feature that automatically stops the recording after 10 minutes was included to enable one-click recording. Once video recording is completed, parents can upload it directly or save it on the device to review the video first before uploading it.

These features were implemented based on results from the first 4 participating families of the controlled experiment. Before these features were implemented in NODA smartCapture, the first 4 participating parents gave an average ease-of-use rating of 3 on a 5-point scale, and the number (4 recordings, 1/scenario) and length of recorded videos (maximum 10 minutes) were not consistent with the instructions that were given. Once these features were implemented, the next set of 4 parents gave an average ease-of-use rating of 4, which was also maintained in the field evaluation. The second set of 4 parents who participated in the controlled experiment and the 5 parents who participated in the in-field evaluation all collected the right number of videos of appropriate length according to the given instructions. In addition, the log analysis confirmed that parents were able to use NODA smartCapture easily during the in-field evaluation. Parents did not rely much on the help menu even without any prior training for using NODA smartCapture. Log analysis showed that only 2 families of the 5 accessed the help menu, 1 and 2 times, respectively. In addition, on average 73% (22/30) of the time, parents took the shortest path from selecting a recording scenario to starting a recording. Because there could be reasons other than complexity of the NODA smartCapture that can contribute to stopping a recording and not completing it, analysis of the workflow focused only on scenario selection to starting a recording.

### Clinical Utility of Video Evidence

Based on data analysis from the controlled experiment in a home-like setting in Stage 2, we identified two key features that increase the clinical utility of the recorded videos. These include an embedded prescription feature and a notification feature.

While rating the clinical utility of videos recorded by parents in the controlled experiment, the collaborating diagnostician identified two sets of issues that negatively influenced utility. The first set related to the set up of the recordings. The most common set-up-related issue was incorrect field of view. For example, parents often captured videos where the face of the child with autism, the relevant toys, or the person that the child was interacting with were not clearly visible on camera, either because of the way the camera was set up or mounted, or because it was too zoomed in or zoomed out. In some cases, parents followed the child around while holding the camera, which was both distracting to the child and prevented the parent from actively playing with the child during the recording. Other times, parents would not set up the camera in advance of recording and would start recording while they are still setting up the camera on the mounting device. The second set of issues related to the frequency and quality of interaction between the child and the parent. Some parents interacted with the child excessively, preventing the clinician from observing what the child does naturally when left alone. Other times there was insufficient interaction between the parent and the child, and the diagnostician wished to observe how the child might react to the parent’s attempts to interact with him/her or whether the child would direct his/her attention to something. Thus, both excessive and insufficient interaction between the parent and the child can make it difficult for a diagnostician to reliably assess the child’s level of functioning.

In response to these two sets of issues, and in consultation with the collaborating diagnostician, we embedded explicit instructions within the NODA smartCapture interface. This clinical prescription (Figure 2) included specific instructions for the parent about how to set up and frame each recording (staging), and how to interact with the child during the recording (social presses). These instructions were intended to maximize the likelihood that the parent records the right kind of video evidence of their child’s behavior from the diagnostician’s perspective. For each of the 4 recording scenarios, we established a set of directions to improve the staging of the recording and a set of social presses that the parent was asked to present to the child. Staging instructions covered the set up of the camera and the environment, such as (1) making sure the child’s face and relevant objects and social partners are in the field of view of the camera; (2) suggestions for appropriate play items, such as toys and books; and (3) across all scenarios, parents were asked to set up the camera ahead of time, and to use a mounting device (tripod). Instructions for social presses included specific actions the parent needed to take during the recording, such as calling the child’s name, pointing to an object to see whether the child will look toward it. These actions represented the types of social presses that a diagnostician might use while assessing the child in person.

In addition to the explicit directions embedded within the NODA smartCapture interface, we realized (through our discussions with the diagnosticians) that diagnosticians may want to guide parents during the in-home recording process*.* For example, the diagnostician may wish to ask the parent to rerecord a scenario because the lighting conditions were poor, or because they want the parent to try a social press again. Therefore, a notification system was included in the system (added before the in-field evaluation) whereby the diagnosticians could send notifications to NODA smartCapture from the NODA Connect Web portal. This feature was not intended to support real-time messaging; rather, it was intended to enable the diagnosticians to review the videos uploaded by a parent for appropriateness in advance of the video being used for the diagnostic assessment, and ask for additional recording as needed.

After incorporating these prescription and notification features, the clinical validity ratings of the parent-collected videos increased from 81% (13/16) in the experimental controlled setting to 96% (26/27) in the in-field evaluation. In total, 10 notifications were sent to families during the field study. Six of these notifications were about instructions for parents to include particular social presses and 4 messages were about confirming the status of recording. Utilization of the notification system reflects its usefulness. Furthermore, the participating diagnosticians in the field evaluation were also asked to rate the usefulness of the videos after they completed remote diagnostic assessment on a 5-point scale (1 indicates “not useful” to 5 indicates “very useful”). Diagnosticians’ rating and qualitative feedback during the follow-up interview confirmed that the videos collected by parents during the in-field evaluation were clinically useful (average rating of 4) for conducting remote diagnostic assessment.

### Completion of Diagnostic Assessment via NODA Connect

The iterative design process of NODA Connect in Stage 2 helped finalize features that support diagnosticians in completing a diagnostic assessment based on the videos recorded by parents. These include the following: (1) a set of predefined tags that the diagnostician can use to flag specific child behaviors in the videos; (2) an integrated DSM checklist where each tag assigned by a clinician is mapped to the relevant DSM subcriterion; and (3) access to the child’s developmental history entered by the parent into the system.

**Figure 3:**
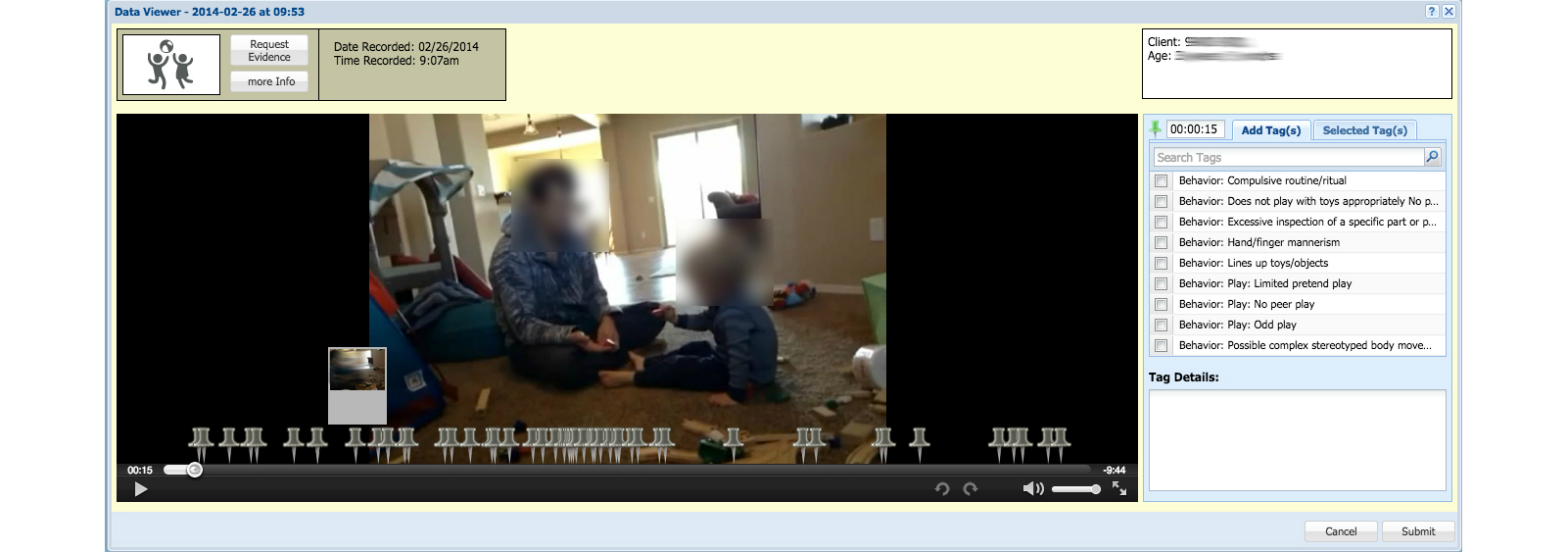
NODA Connect: Web-based assessment portal video tagging.

Once diagnosticians receive appropriate video recordings, they can review them and begin tagging them with behaviors relevant to diagnosing autism (Figure 3). The NODA Connect has a built-in set of tags representing specific behavioral markers such as “no eye contact” or “repetitive play,” which were created based on the diagnostic criteria for autism within the DSM. The list of tags was compiled by the collaborating diagnostician and the autism domain expert, and vetted through conversations with several other clinical experts. In total, there were 66 tags included in NODA Connect. These tags included both atypical (n=57) behavior tags (representing atypical development) and typical (n=9) behavior tags (representing typical development). The goal of the tagging step is to have the diagnostician watch the videos for any evidence of atypical or typical behavior, and flag moments in time when that behavior occurs, without yet considering specific DSM criteria.

**Figure 4:**
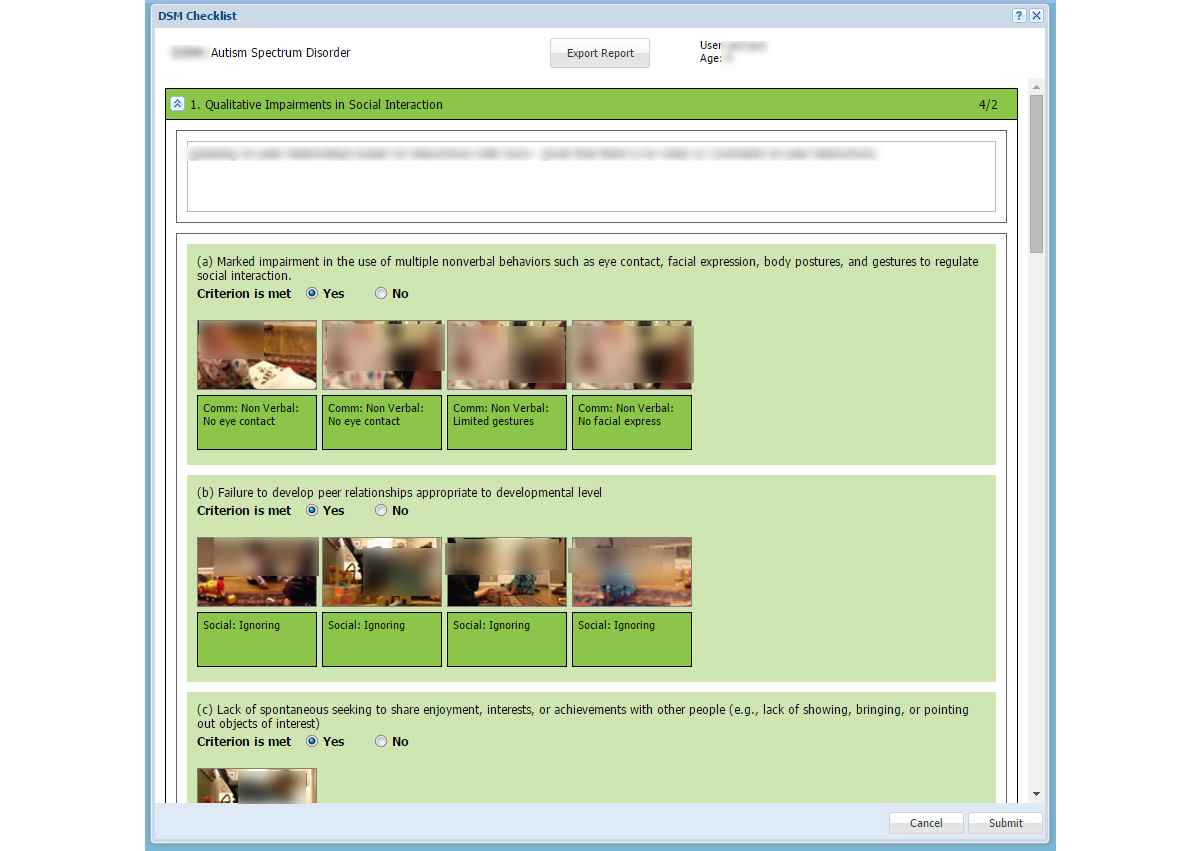
NODA Connect: Web-based assessment portal showing the DSM checklist screen.

Once all the videos for a child are viewed and tagged, the diagnostician can review the DSM diagnostic checklist (Figure 4). At the time this research study was conducted, the DSM-IV was still in use, and thus, formed the basis of the diagnostic checklist in the NODA Connect assessment portal. Subsequent to the release of the DSM-V, tags and the diagnostic checklist were updated to reflect the new framework.

The DSM checklist contains categories of symptoms, with specific subcriteria for each category. The DSM-IV included the following diagnostic categories: (1) qualitative impairments in social interaction; (2) qualitative impairments in communication; and (3) restricted, repetitive and stereotyped patterns of behavior, interests, and activities. Each of these 3 categories included 4 subcriteria. Within NODA Connect, each tag inserted in the videos by the diagnostician during the video review step is automatically mapped to the relevant subcriterion, and shows up as a video snippet (Figure 4).

Within the DSM checklist, the diagnosticians can review the tags and then check a Yes/No box to indicate whether, based on the tags and their clinical judgment, the child meets that specific criterion. Once the entire DSM checklist is filled, the diagnostician makes a determination about the child’s diagnosis of autism based on the DSM criteria, developmental history, and clinical judgment. Note that within NODA Connect the diagnosticians have access to the child’s developmental history that parents fill during the in-home video-collection phase, although during our in-field evaluation we restricted access to this checklist so that diagnosticians would remain blind to the child’s true diagnostic status.

Results from the in-field evaluation confirmed that NODA Connect features support diagnosticians in completing a diagnostic assessment. Overall, in 91% of assessments (10/11) via NODA Connect, diagnosticians reached a decision about diagnostic outcome that matched with the child’s previous diagnostic status.

Analysis of the NODA Connect usage pattern during the in-field evaluation showed that there was not much variability in the time taken to complete tagging and filling the DSM checklist by different diagnosticians. Across all assessments, the total time taken to complete tagging all the videos of a child and filling out the DSM checklist was on average 62 minutes (SD 14.8 minutes). Diagnosticians spent the least amount of time (average 37 minutes) completing the assessment for the child who was typically developing. After removing the two assessments for this child from the analysis, the average time spent tagging videos and completing the DSM checklist was even more consistent (average 68 minutes, SD 8.3 minutes).

### Comparative Results of Diagnostic Outcomes

The main focus of the work presented here was to iteratively develop and evaluate the design of the two systems, NODA smartCapture and NODA Connect. In addition, given that during the in-field evaluation the diagnosticians assigned a diagnosis to the child upon completing the assessment, we were able to compare the diagnosis conducted through NODA Connect with the child’s previous diagnosis as indicated in the child’s medical record. For 4 of the 5 children (3 children with a previous diagnosis of autism and 1 typically developing child), both the remote diagnosticians independently arrived at the same diagnostic decision, and in agreement with the child’s actual diagnostic status. For the fifth child (with a previous autism diagnosis), one diagnostician matched the diagnosis in the child’s record but the other did not, although the latter indicated with high confidence that the child was not typically developing. A third diagnostician independently reviewed this case via NODA Connect and also confirmed the diagnosis in the child’s medical record. Overall, in 91% of assessments (10/11) via NODA Connect, diagnosticians reached a decision about diagnostic outcome that matched with the child’s previous diagnostic status.

## Discussion

### Principal Findings

The iterative design approach undertaken in this study enabled us to identify specific features of a store-and-forward telehealth platform that supports remote diagnosis of autism using videos recorded by families in their homes. The results of the in-field evaluation of NODA smartCapture and NODA Connect demonstrated that our system design allowed parents to easily capture clinically useful evidence of child behavior, and diagnosticians to complete a diagnostic assessment of autism with high confidence. See Multimedia Appendix 1 for the most recent version of NODA Capture and Connect resulted from this work.

This section discusses the perspectives of the various stakeholders on the perceived utility and limitations of the system, potential design enhancements, our vision for the large-scale adoption of the system within current autism diagnostic practices, and how our prescription, collection, and assessment model can be generalized to other clinical assessment applications.

### Perceived Utility and Limitations

During initial stakeholder interviews in Stage 1, parents and clinicians considered the concept of video collection and sharing of in-home behavioral evidence potentially valuable for a variety of reasons. They reported that this approach can allow clinicians to observe otherwise inaccessible behaviors (eg, less-frequent behaviors, behavior triggers at home) in their natural context, and to view family-child interactions. Moreover, it can efficiently connect parents and clinicians for timely assessment of the child as, unlike current practice, clinicians can have immediate access to the behavior evidence. However, during the same interviews, parents and clinicians also highlighted several potential barriers to the adoption of an in-home video-recording system. The most commonly mentioned concerns were system complexity, privacy concerns, and child’s reactivity. Parents suggested that having explicit data capture and sharing policies, and control over data collection and sharing would alleviate privacy concerns. They also indicated that they would be willing to sacrifice some privacy concerns to get help with a more timely diagnosis for their child. Parents and clinicians also highlighted that the recording device may cause the child to react differently than he or she would otherwise. However, clinicians reported that for them, the child’s reactivity to being recorded would not necessarily invalidate the clinical utility of the video evidence, as such reactivity happens during clinic-based observations as well. Parents and clinicians appreciated that the recording application could be installed on mobile phones and tablets because these are everyday objects that children are used to seeing and the reactivity effects would thus likely be minimal*.*

During the in-field evaluation, diagnosticians appreciated that the system helped them conduct an autism diagnosis based on naturalistic behavioral evidence. They also highlighted that, unlike direct observation, video observation would allow them to go back in time to review and verify certain observations, if required. Among the 3 participating diagnosticians in the in-field evaluation, 2 had no previous experience with video observation. These 2 reported that before the study they were reluctant and skeptical about the value of in-home video recording for diagnostic assessment. The third diagnostician had previously participated in other research efforts that involve video observation for assessment and interventions of children with autism and had previously found these methods valuable. However, all the diagnosticians, irrespective of their initial biases, reported that using the remote diagnosis system left them feeling it was extremely valuable and effective for remote autism diagnosis. However, the diagnosticians also identified potential situations when in-home behavior evidence along with a brief developmental history may not be sufficient to complete a diagnostic assessment of autism. These situations included (1) when the child is too young (<2 years old); (2) when the child has very subtle characteristics of autism; and (3) when the child’s level of functioning is very limited. According to the diagnosticians, in all these cases it may be difficult to make a judgment about the child’s overall development level, which is required for comparison with the child’s social profile. In such cases, supplementary evidence in addition to video evidence would be required, which, depending on the situation, could be a parent report, a standard developmental assessment, or even direct observation of the child.

### Technology Enhancements

Advanced technology features can be incorporated into the existing NODA smartCapture and NODA Connect for built-in intelligence. For example, there are a number of factors associated with staging (lighting conditions, audio quality, field of view, whether the child’s face is in view, etc) that a recording system could automatically detect during the recording and alert parents to rerecord without the need for the diagnostician to review the videos first. In addition, results from the in-field evaluation indicated that on average tagging videos took 84.6% (52.5/62 minutes) of the total time spent on completing 1 diagnostic assessment via NODA Connect. The amount of time spent on tagging could be significantly reduced if the Web-based assessment system were to include an automated tagging process. For example, certain detectable behaviors such as response to name call, a smile, giving or taking an object, eye contact, stereotypical behaviors are reasonable candidates to be automatically detected within collected video evidence, given the recent advances in automated video analysis [33-35]. Because these recognition techniques would not be perfect, the system could suggest potential tags in the video timeline and allow the diagnosticians to confirm or reject them. As another example, the assessment system could learn the diagnostician’s tag assignment behaviors and highlight the most frequently assigned tags so the diagnostician could quickly locate them.

### Diagnostic Workflow and Field Adoption

An open question for future research is to explore a feasible workflow for wide-scale adoption of our remote diagnostic system. One workflow that we envision involves a referral mechanism for remote diagnostic assessment like any other laboratory tests. Pediatricians are often the first medical professionals to identify children as potentially showing early signs of autism, and are responsible for referring families to a specialist for further assessment. In the proposed workflow, the pediatrician can refer the family for a remote diagnostic assessment. Upon connecting with the remote assessment service, the parents download NODA smartCapture directly to their mobile phone. A diagnostician at an affiliated diagnostic center can then guide the in-home evidence-collection procedure and complete the diagnostic assessment through NODA Connect. Finally, an electronic diagnostic report summarizing diagnostician’s video observation, DSM checklist, and diagnostic outcome can be shared with the pediatrician, who then shares it with parents.

Overall, this workflow has two potential benefits. First, it engages pediatricians, which is beneficial because research suggests that pediatrician involvement in the referral and diagnostic process can result in more timely diagnosis [23,36-39]. A pediatrician sees children at regular intervals during the early years of development and is in the best position to note early warning signs and take appropriate timely action. Second, this workflow model may allow autism diagnostic centers to serve more families by remotely assessing children for the purposes of triage. Children whose diagnostic outcome is not clear through this remote procedure can be seen in person for a more comprehensive diagnostic assessment.

### Generalizability of Our Approach

Our remote diagnostic assessment system is based on a prescription, behavior specimen collection, and assessment model. Analogous to traditional medical specimen collection and assessment process, this model involves (1) a clinician’s prescription for behavior specimens (in the form of short videos) to be collected; (2) in-home collection of behavior specimens by parents; and (3) the assessment of behavior specimens by a remotely located clinician.

This is a generic model that is transferable, beyond remote autism diagnosis, to other clinical situations where an analysis of behavior by a professional is key to the clinical assessment. Any condition or situation in which observation of behavior in the natural environment is of value, and for which those behaviors can be specified to ensure relevant examples are recorded, is a candidate use case. During stakeholder interviews, the participating clinicians suggested a number of potential use cases where this model could be applicable and valuable. One such use case is to sort and prioritize families on waiting lists for clinical services to expedite the intake process. Sorting and prioritizing the waiting list is crucial, because timely access to diagnostic and intervention services is often hampered by long waiting lists at centers and clinics. In addition, a system based on such a model may be valuable for providing treatment and follow-up services to remotely located patients who do not have easy access to the clinic. Another use case is parent training, such as those involving clinicians training parents to implement an intervention at home.

Although the prescription, collection, and assessment model along with its high-level design features (embedded prescription, guided capture through notification feature, tagging, video observation, assessment based on mapped tags) are both generic, it must be customized within the context of its end-use-case scenario. For instance, the embedded prescribed instructions in the recording application can be contextualized through a new prescription writing pad feature within the Web-based assessment portal. One example of successful transfer and customization of our approach is the use case of medication management. In-home behavior specimens captured and shared through the mobile phone-based system allow physicians to monitor medication side effects and note any improvements in symptoms between office visits using the Web-based assessment portal. In a preliminary evaluation, physicians highlighted that this medication administration system assisted them in monitoring patients with autism spectrum disorder more comprehensively and accurately than using subjective reports provided by caregivers during office visits [40].

### Conclusions

The in-field evaluation demonstrated that the system’s design enabled parents to easily record clinically valid evidence of their child’s behavior, and diagnosticians to complete a diagnostic assessment for autism. These results shed light on the potential for appropriately designed telehealth technology to support clinical assessments using in-home video captured by families. This assessment model can be readily generalized to other conditions where direct observation of behavior plays a central role in the assessment process.

The results of this paper are not a final statement on the clinical validity of diagnostic outcome; rather, this paper reports on the design of the remote autism diagnosis system that resulted from an iterative design process and has shown a promising conclusion from an evaluation in the field. The next step is to validate the diagnostic outcome through a clinical trial in which a large sample of children would be assessed via both remote autism diagnosis system and standard in-person diagnostic assessments for comparison.

## Appendix

Multimedia Appendix 1 The most recent version of NODA smartCapture and Connect.

## Abbreviations

DSM: Diagnostic and Statistical Manual of Mental Disorders
NODA: naturalistic observation diagnostic assessment
