# Supplementary material for: A Novel System for Supporting Autism Diagnosis Using Home Videos: Iterative Development and Evaluation of System Design
Source: JMIR Mhealth Uhealth. 2015 Jun 17;3(2):e68. doi: 10.2196/mhealth.4393 (PMC4526946; doi:10.2196/mhealth.4393)
Supplement: Multimedia Appendix 1 [file mhealth_v3i2e68_app1.pdf]

NODA smartCapture and Connect

(Most recent version walkthrough)

- **NODA smartCapture**

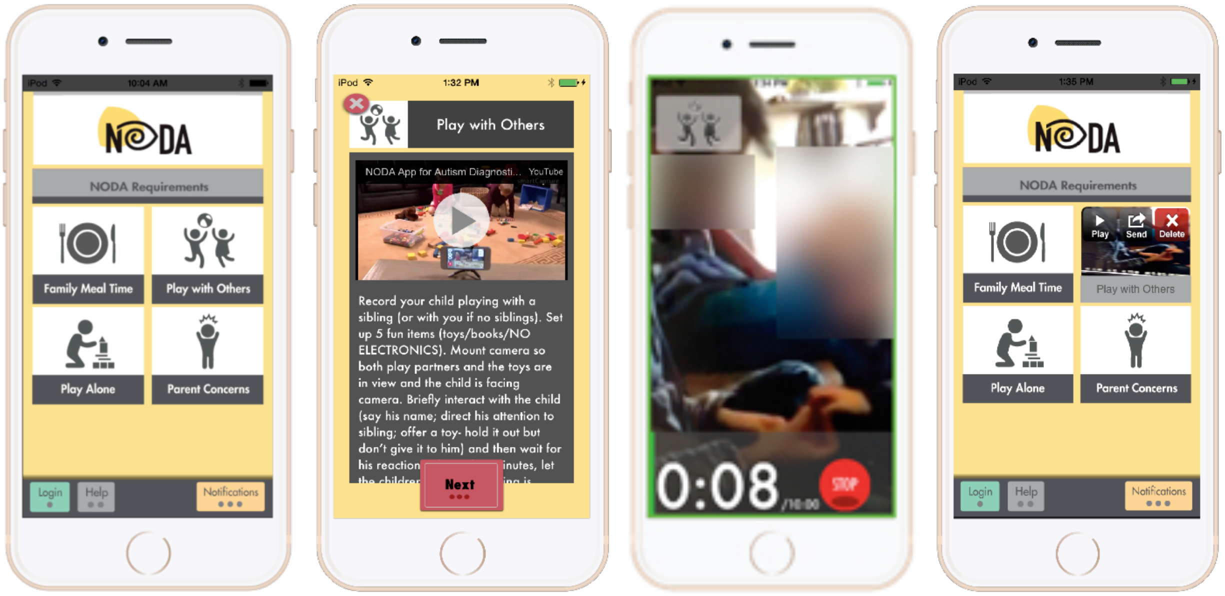

a. *b.* *c.* *d.*

Figgure 1: a) Home screen showing four NODA scenarios b) Each scenario has embedde precription that includes a 'sample video' and recording instructions for parents c) Recording mode 4) Home screen showing status of the ones recorded.

Sample videos that are embedded within NODA

- **Meal time:** https://www.youtube.com/watch?v=d40_rDAqprg
- **Play alone:** https://www.youtube.com/watch?v=4Clh027Zyo0
- **Play with other:** https://www.youtube.com/watch?v=z3oKRoGOlac
- **Parents concern:** https://www.youtube.com/watch?v=nAyw6GOUMlc

- **NODA Connect** (HIPAA compliant)

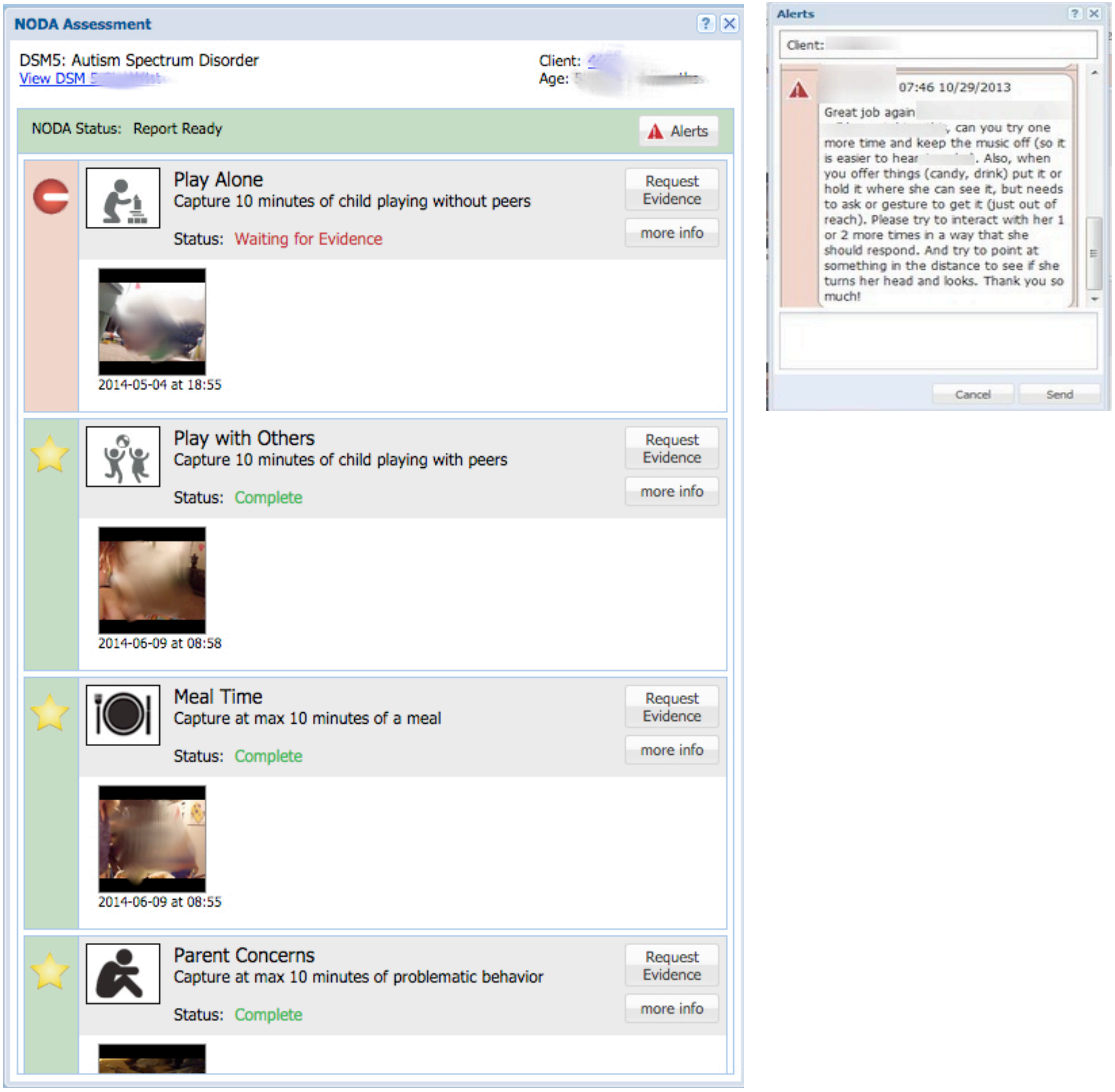

Figure 2: Upon selecting a family, the diagnostician can see which scenarios have been uploaded and which remain to be uploaded. The diagnostician can send an alert to the NODA SmartCapture for requesting parents for additional recordings.

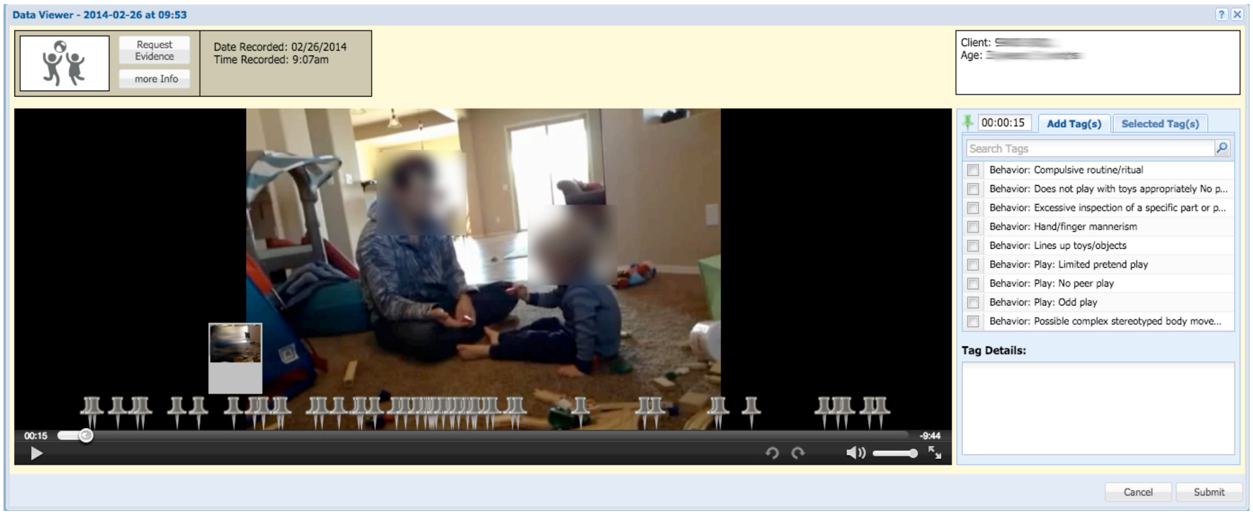

Figure 3: The diagnostician can review videos and assign tags.

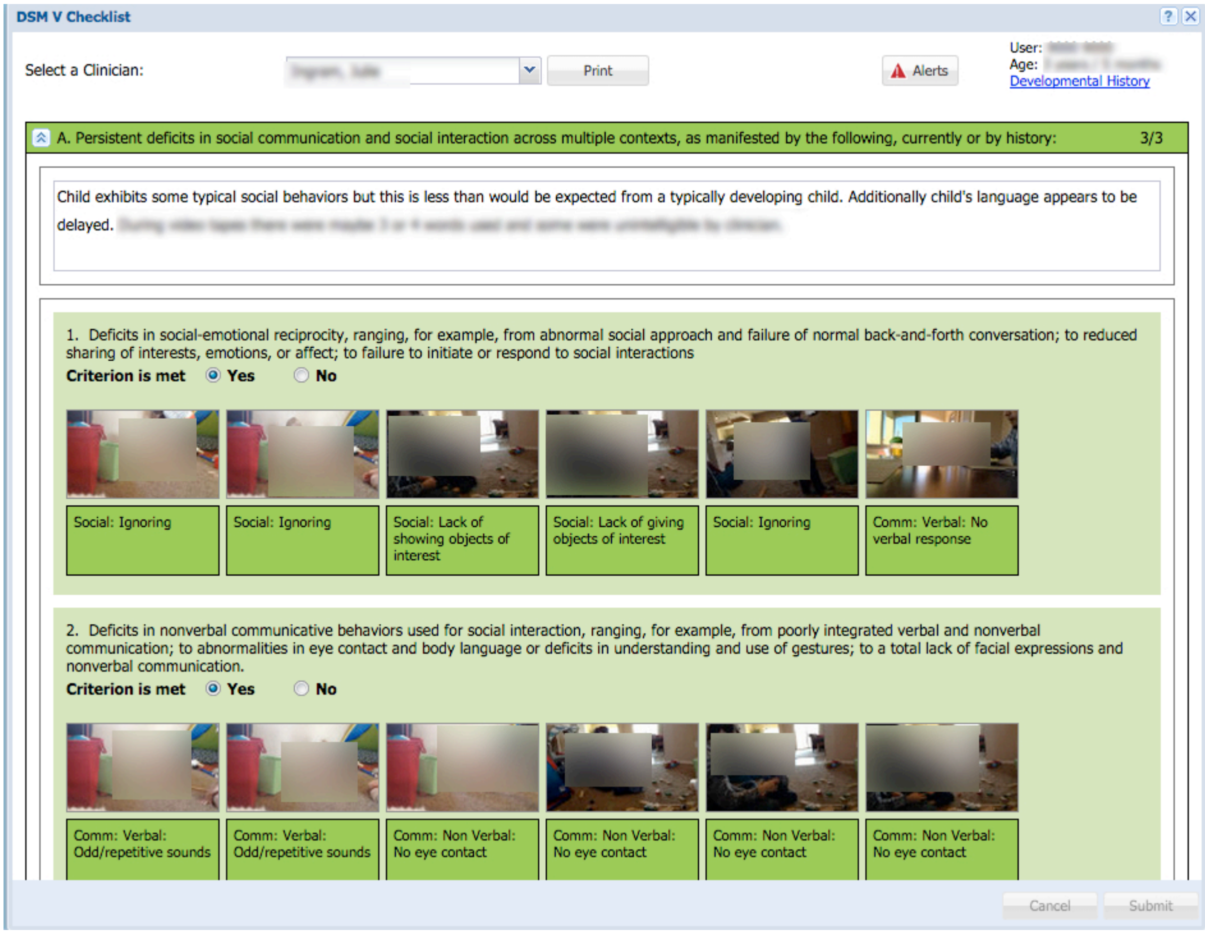

Figure 4: All tags inserted into videos are automatically linked to the associated DSM-5 criteria. The diagnostician can review the tags associated with criteria and fills DSM checklist.

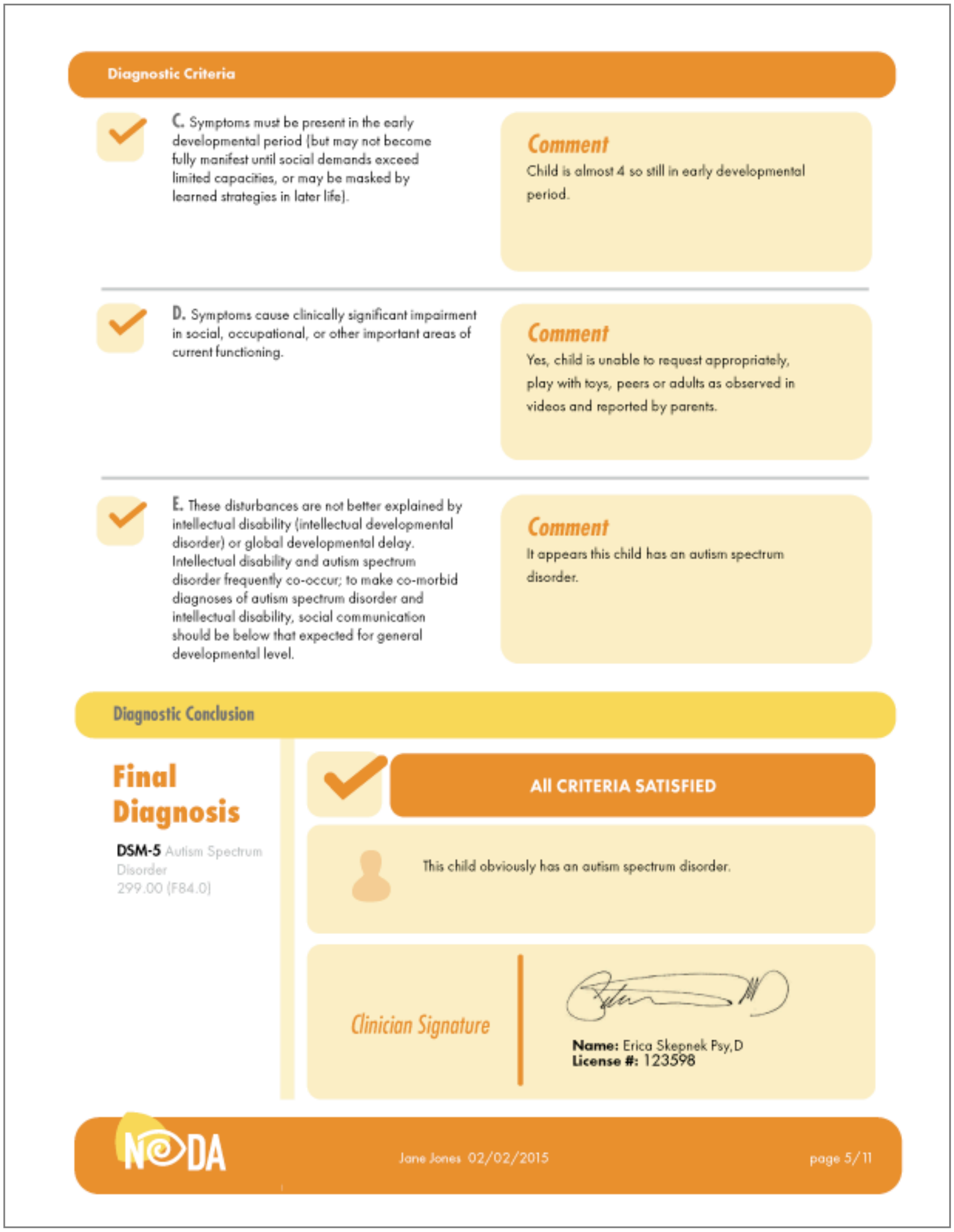

Diagnostic Criteria

C. Symptoms must be present in the early developmental period (but may not become fully manifest until social demands exceed limited capacities, or may be masked by learned strategies in later life).

Comment
Child is almost 4 so still in early developmental period.

D. Symptoms cause clinically significant impairment in social, occupational, or other important areas of current functioning.

Comment
Yes, child is unable to request appropriately, play with toys, peers or adults as observed in videos and reported by parents.

E. These disturbances are not better explained by intellectual disability (intellectual developmental disorder) or global developmental delay. Intellectual disability and autism spectrum disorder frequently co-occur; to make co-morbid diagnoses of autism spectrum disorder and intellectual disability, social communication should be below that expected for general developmental level.

Comment
It appears this child has an autism spectrum disorder.

Diagnostic Conclusion

Final Diagnosis

DSM-5 Autism Spectrum Disorder
299.00 (F84.0)

All CRITERIA SATISFIED

This child obviously has an autism spectrum disorder.

Clinician Signature

Name: Erica Skepnek Psy,D
License #: 123598

NODA Jane Jones 02/02/2015 page 5/11

Figure 5: A diagnostic report is generated that summarizes the evidence to support each DSM criterion and the diagnostician's clinical judgment. The report can be shared with the parent and the referring pediatrician.
